# DWI-Positive Lesions in Patients With Acute Intracerebral Hemorrhage: Associations With Cerebral Small Vessel Disease and Clinical Outcome

**DOI:** 10.1212/WNL.0000000000218449

**Published:** 2026-08-14

**Authors:** Martina Locatelli, Hatice Ozkan, Giulia Avola, Philip S. Nash, Gareth Ambler, Simon Fandler-Höfler, Yang Du, Wenpeng Zhang, Robert J. Simister, David John Werring, Hans Rolf Jäger

**Affiliations:** 1 Stroke Research Centre, Department of Translational Neuroscience and Stroke, UCL Queen Square Institute of Neurology, London, United Kingdom;; 2 Comprehensive Stroke Service, National Hospital for Neurology and Neurosurgery, University College London Hospitals NHS Trust, UCL Queen Square Institute of Neurology, United Kingdom;; 3 Department of Medicine and Surgery, University of Parma, Italy;; 4 Department of Statistical Science, University College London, United Kingdom;; 5 Department of Neurology, Medical University of Graz, Austria; and; 6 Neuroradiological Academic Unit, UCL Queen Square Institute of Neurology, London, United Kingdom.

## Abstract

**Background and Objectives:**

Diffusion-weighted imaging (DWI)-positive lesions are increasingly recognized as markers of cerebral small vessel disease (cSVD) in acute intracerebral hemorrhage (ICH), but their underlying mechanisms, clinical-radiologic associations, and prognostic significance remain uncertain. We therefore aimed to determine their prevalence, anatomical distribution, and associations with baseline clinical-radiologic features and 6-month functional outcome assessed by the modified Rankin Scale (mRS).

**Methods:**

In this prospective cohort study, we analyzed consecutive patients with ICH included in the Stroke Investigation Group in North and Central London registry. We assessed the presence and anatomical distribution of DWI-positive lesions on acute MRI, as well as other cSVD markers rated according to the STandards for ReportIng Vascular changes on nEuroimaging criteria. The primary outcome was disability at 6 months assessed using the mRS. We investigated associations between DWI-positive lesions, clinical-radiologic features, and poor 6-month functional outcome (mRS ≥3) using adjusted multivariable logistic regression.

**Results:**

We included 342 patients (median age 67 years [interquartile range (IQR) 55–78]; 142 (41.5%) women; 138 (40.3%) lobar ICH; median cSVD burden 2 [IQR 1–3]). Overall, 74 patients (21.6%) had DWI-positive lesions, most commonly in lobar regions (33 [44.6%]). Patients with cerebral amyloid angiopathy (CAA) more frequently had strictly lobar DWI-positive lesions compared with those without (78.6% vs 36.7%); strictly deep DWI-positive lesions were exclusively observed in the non-CAA group (25.0% vs 0%). Compared with patients without DWI-positive lesions, those with DWI-positive lesions more frequently had severe white matter hyperintensities (WMH; Fazekas ≥2: 81.1% vs 64.2%, *p* < 0.001), higher cerebral microbleed (CMB) burden (median = 3 vs 1, *p* = 0.034), and larger ICH volume (median = 14 mL vs 7 mL, *p* = 0.0006). In adjusted analyses, DWI-positive lesions were independently associated with severe WMH (odds ratio [OR] 3.37, 95% CI 2.72–6.89), ICH volume ≥30 mL (OR 2.29, 95% CI 1.76–3.42), CMBs (OR 1.61, 95% CI 1.21–2.93), and poor functional outcome at 6 months (OR 2.16, 95% CI 1.67–3.48).

**Discussion:**

DWI-positive lesions are common after acute ICH and are independently associated with cSVD and poor functional outcome, supporting their role as a prognostic biomarker. Their anatomical distribution differs according to the underlying cSVD subtype.

## Introduction

Spontaneous intracerebral hemorrhage (ICH) accounts for 10%–15% of strokes in Western populations and 18%–20% in Asian populations^1-3^ yet causes disproportionately severe long-term disability and mortality, with a 1-year survival rate of approximately 40%.^4^ The incidence of ICH has remained largely stable at 10–20 cases per 100,000 person-years,^5,6^ but its prognosis has not substantially improved because of the lack of effective treatments.^7,8^ Over 75% of spontaneous ICHs are attributable to cerebral small vessel disease (cSVD),^9^ primarily arteriolosclerosis or cerebral amyloid angiopathy (CAA).^9-11^

Recent studies have identified the occurrence of small diffusion-weighted imaging (DWI)-positive lesions in a subset of patients with spontaneous ICH, typically within the first days of admission^12^ but also seen at later time points.^13^ DWI-positive lesions are believed to represent small acute ischemic lesions based on their imaging characteristics and have emerged as a marker of cSVD.^14^ Although the overall prevalence of DWI-positive lesions after ICH seems similar across different cSVD subtypes, it remains unclear whether their anatomical distribution could aid in distinguishing between these subtypes.^15^ DWI-positive lesions have been associated with worse functional outcomes after ICH, but most available studies have methodological limitations, including short (3-month) follow-up^16^; a focus solely on patients with ICH attributed to arteriolosclerosis in populations restricted to Korea or China^16-18^; or including populations from randomized controlled trials with inherent selection bias,^19,20^ limiting the generalizability of their findings.

One interpretation is that DWI-positive lesions represent small acute ischemic lesions due to a diffuse active occlusive cSVD associated with impaired cerebrovascular reserve.^13-15^ An alternative, and not mutually exclusive, hypothesis is that DWI-positive lesions could be related to the acute ICH (e.g., neuroinflammation, disturbed autoregulation, and cortical spreading depression), subsequent therapy (e.g., early blood pressure lowering, surgery, or hemostatic therapy), the underlying small vessel arteriopathy that has caused the acute ICH, or a combination of some or all of these factors.^18,19,21^

We therefore hypothesized that the presence of DWI-positive lesions identifies patients with more active occlusive cSVD that is more likely to progress and be associated with worse functional outcome and increased risk of recurrent stroke. To test this hypothesis, we investigated DWI-positive lesions in a prospective stroke registry with a high rate of MRI to determine their prevalence and associations with clinical-radiologic characteristics and functional outcomes measured by the modified Rankin Scale (mRS) at the 6-month follow-up. We also explored whether the anatomical distribution of DWI-positive lesions differed between cSVD subtypes (probable CAA vs non-CAA).

## Methods

### Patient Selection

For this study, we included individual patient data from the Stroke Investigation Group in North and Central London (SIGNAL) registry, which included consecutive patients older than 18 years with imaging-confirmed acute ICH from the North Central London region (population approximately 1.5 million) treated at the University College London (UCL) Hospitals NHS Foundation Trust from January 1, 2017, to January 13, 2020.^22^ SIGNAL included all clinically available baseline acute brain MRI data, detailed clinical data, sociodemographic data (age, sex, ethnic origin, and medical history were collected from the patients' electronic health records), date of ICH admission, and neuroimaging data collected using a structured evaluation form. Individual patient data were validated against electronic health records, and brain imaging data were verified against neuroradiology reports and available imaging scans for the diagnosis of ICH by 3 clinical neurologists.

We included all patients with a diagnosis of ICH and diagnostic quality MRI scans performed during acute hospital admission within 90 days of emergency admission. This imaging time window was chosen to maximize inclusion and minimize selection bias toward patients who were initially too medically unstable to undergo MRI at emergency stroke admission but later underwent MRI during their acute stroke unit stay. We excluded all patients who did not have a diagnostic quality MRI, isolated intraventricular or subarachnoid hemorrhage only, or secondary causes of ICH (including macrovascular or structural lesions).

### Neuroimaging Data

Standardized clinical MRI protocols included at least T1-weighted, T2-weighted, T2-weighted fluid-attenuated inversion recovery (FLAIR), DWI, apparent diffusion coefficient (ADC) maps, and gradient-echo T2* or susceptibility-weighted imaging scans. All included patients had FLAIR sequences. We developed a dedicated rating proforma to record data for each patient. Three clinical neurologists independently rated scans under the guidance of a senior neuroradiologist, achieving good inter-rater and intrarater reliability (κ values >0.75).

DWI-positive lesions were identified on DWI and ADC maps, supplemented with FLAIR, T1-weighted, and T2-weighted sequences, as appropriate, according to the STandards for ReportIng Vascular changes on nEuroimaging (STRIVE-2) criteria.^14^ Only DWI-positive lesions remote from the ICH site were included (remote was considered as lying outside a perimeter of 1 cm from the hematoma), and DWI hyperintense lesions at the margins of the hematoma likely to reflect ICH-related acute tissue injury^12,23^ were excluded. DWI-positive lesions were further classified by location as lobar, deep, or infratentorial, consistent with the anatomical framework of the Microbleed Anatomical Rating Scale (MARS) criteria, and their precise anatomical location (cortex, white matter, basal ganglia, thalamus, brainstem, or cerebellum) was recorded.

ICH location and volume were assessed from the first available brain CT scan or from the earliest available MRI if CT was unavailable. ICH location was categorized using the Cerebral Haemorrhage Anatomical Rating Tool^24^ as lobar, deep, or infratentorial. ICH volume was estimated using the ABC/2 formula. We assessed cSVD markers—including white matter hyperintensities (WMH), lacunes, enlarged perivascular spaces (EPVSs), cortical superficial siderosis (cSS), and cerebral microbleeds (CMBs)—according to the STRIVE-2 criteria.^14^ The distribution of CMB was rated according to the MARS,^25^ WMH were graded using the simplified Fazekas score (0–3),^26^ and cSS was rated as disseminated when affecting >3 sulci. A combined total SVD burden score^27^ was calculated as graded from 0 to 4 depending on the presence of moderate-severe WMH (presence defined as deep Fazekas score ≥2 or periventricular score ≥3), ≥1 lacune, ≥1 microbleed, and basal ganglia enlarged perivascular spaces (BGPVSs) graded ≥2.

ICH etiology was determined according to a modified version of the Clinical and Radiological Analysis of Spontaneous Intracerebral Haemorrhage classification.^28^ We classified arteriolosclerosis as nonlobar ICH accompanied by at least one of the following: ≥1 lacune, moderate-to-severe WMH, ≥1 deep (including brainstem) CMBs, or severe BGPVSs (defined as ≥21 PVSs) without evidence of lobar CMBs or cSS. Probable CAA was diagnosed based on the Boston 2.0 criteria for probable CAA.^29^ Cases were classified as cryptogenic when no MRI-visible signs of SVD were detected.

### Six-Month Outcome Data

The primary follow-up outcome was functional disability, assessed using the mRS, by clinically trained practitioners at 6 months after ICH. We dichotomized mRS as the favorable outcome (mRS 0–2), indicating functional independence (i.e., patients able to look after their own affairs without assistance), and unfavorable outcome (mRS 3–6), indicating dependence or death (i.e., patients requiring assistance with daily activities, bedridden, or dead).

We also assessed recurrent stroke through review of all available electronic health records and regional hospital care systems. All suspected recurrent strokes were adjudicated by trained neurologists and neuroradiologists through detailed case-by-case review with neuroimaging confirmation.

To maximize follow-up and minimize patient burden, participants were offered multiple options for outcome assessment, including attendance at local stroke outpatient clinics, telephone interview, home visit, or postal questionnaire. Additional supportive measures were implemented to facilitate participation. For patients with communication difficulties, cognitive impairment, language barriers, or severe disability (mRS 4–5) that prevented clinic attendance, follow-up was conducted using extended interviews, telephone assessments, or postal questionnaires.

### Statistical Analyses

Sociodemographic, clinical, and neuroimaging characteristics were described using frequency (%) for categorical variables and as means (SD) or medians (interquartile range [IQR]) for continuous variables, according to data distribution. We first calculated the overall prevalence of DWI-positive lesions for the study population. Patients were then stratified according to the presence of DWI-positive lesions (DWI+ vs DWI−) and clinical outcome, categorized as favorable (mRS 0–2) or unfavorable (mRS 3–6) prognosis. Comparisons between groups were performed for demographic, clinical, outcome, and neuroradiologic variables using the χ^2^ test for categorical variables and the Student *t* test and Wilcoxon-Mann-Whitney test for continuous variables, according to distribution. We investigated DWI-positive lesion prevalence and location across different ICH etiologic subgroups (CAA vs non-CAA).

Multivariable logistic regression analyses were performed to identify variables independently associated with the presence of DWI-positive lesions and with poor mRS at 6 months. Variable selection was performed using the Least Absolute Shrinkage and Selection Operator method, which automatically identifies variables independently associated with the outcome while preventing overfitting. Models included factors associated with the outcome at *p* < 0.10 in univariable analyses. Clinically relevant covariates, including age, sex, and stroke severity, were retained in all final models, irrespective of their statistical significance, to ensure adequate adjustment for biologically plausible confounders. The results from the final models were reported as odds ratios (ORs) with corresponding 95% CIs.

All analyses were performed using STATA/MP version 18 (StataCorp LLC, College Station, TX), and statistical significance was set at *p* < 0.05.

### Standard Protocol Approvals, Registrations, and Patient Consents

The SIGNAL registry was approved by the UCL Hospitals NHS Foundation Trust Governance Review Board (code: 5-201920-SE) as a continuous service evaluation of the comprehensive clinical care program and London South-East REC (24/LO/0368), providing permission to use data collected during the routine care of patients with ICH. Because this was a prospective cohort study using only routinely collected clinical data, the need for individual informed consent was waived. This study was written in accordance with the Strengthening the Reporting of Observational Studies in Epidemiology reporting guidelines.

### Data Availability

The corresponding author will consider requests to access fully anonymized data included in this study, but these will be subject to UCL Hospitals NHS Foundation Trust Governance Review Board approval.

## Results

### Baseline Characteristics

The patient selection process is shown in eFigure 1. Between 2017 and 2020, 547 patients with ICH were admitted to our stroke service; 205 were excluded because of missing MRI data (143 patients with CT-only imaging because of contraindications to MRI, stroke severity, dementia, or clinical instability; 29 without DWI sequences; and 33 with secondary causes of ICH, such as brain tumors) or not meeting the eligibility criteria; the final cohort comprised 342 patients. The patient characteristic data for excluded patients are presented in eTable 1. In brief, patients who were excluded were significantly more likely to be older; have higher rates of AF diagnosis, heart failure, and moderate stroke severity; and had longer length of hospital stay.

The sociodemographic and clinical characteristics of the 342 included patients are summarized in Table 1. The median (IQR) age was 67 years (55–78), with 41.5% female, and 53.8% of patients were White; 74 had DWI-positive lesions (21.6%). MRI was performed at a median of 4 days after acute ICH (IQR 2–9). Time from hospital admission to MRI acquisition did not differ between patients with and without DWI-positive lesions; in both groups, MRI was performed at a median of 4 days. A history of stroke/TIA (21.6% vs 15.7%), smoking (31.1% vs 6.7%), and alcohol consumption (14.9% vs 2.6%) was more common among patients with DWI-positive lesions. On the other hand, patients with DWI-positive lesions were less commonly female (31.1% vs 44.4%).

**Table 1:** Unadjusted Sociodemographic and Clinical Patient Characteristics

Variables	All (N = 342)	DWI+ (N = 74)	DWI− (N = 268)	*p* Value
Age (y), median (IQR)	67 (55–78)	63 (51–87)	66 (54–89)	0.824
Female sex, n (%)	142 (41.5)	23 (31.1)	119 (44.4)	0.040^a^
Ethnic origin, n (%)				
White	184 (53.8)	36 (48.6)	148 (55.2)	0.539
Black	49 (14.3)	11 (14.9)	38 (14.2)	—
Asian	40 (11.7)	9 (12.2)	31 (11.6)	—
Others	69 (20.2)	18 (24.3)	51 (19.0)	—
Clinical characteristics				
Hypertension, median (IQR)	208 (60.8)	42 (56.8)	166 (61.9)	0.419
SBP admission (mm Hg), median (IQR)	174 (143–200)	172 (149–196)	175 (153–200)	0.811
Previous stroke/TIA, n (%)	58 (17.0)	16 (21.6)	42 (15.7)	0.027^a^
Heart failure, n (%)	4 (1.2)	1 (1.3)	3 (1.1)	0.879
AF, n (%)	35 (10.2)	7 (9.5)	28 (10.4)	0.804
Diabetes, n (%)	53 (15.5)	11 (14.9)	42 (15.7)	0.865
Smoking, n (%)	41 (12.0)	23 (31.1)	18 (6.7)	0.004^a^
Alcohol, n (%)	18 (5.3)	11 (14.9)	7 (2.6)	0.023^a^
Admission NIHSS score, median (IQR)	6 (4–12)	9 (4–1)	7 (3–11)	0.092
Admission to MRI (d), median (IQR)	4 (2–9)	4 (2–10)	4 (2–9)	0.9300
Length of hospital stay (d), median (IQR)	4 (2–7)	4 (2–10)	3 (2–7)	0.1627
Clinical outcomes				
Discharge mRS, median (IQR)	4 (3–5)	3 (1–4)	2 (1–4)	0.018^a^
6-months mRS (N = 332), median (IQR)	3 (2–5)	3 (2–5)	2 (1–3)	<0.001^a^
Death (N = 332), n (%)	23 (6.7)	10 (13.5)	13 (4.8)	0.062
Recurrent stroke, n (%)	32 (9.4)	15 (20.3)	17 (6.3)	0.037^a^
Ischemic stroke, n (%)	14 (4.1)	5 (6.8)	9 (3.4)	0.016^a^
Recurrent ICH, n (%)	18 (5.3)	10 (13.5)	8 (3.0)	0.001^a^

Abbreviations: AF = atrial fibrillation; DWI = diffusion-weighted imaging; ICH = intracerebral hemorrhage; IQR = interquartile range; mRS = modified Rankin Scale; NIHSS = NIH Stroke Scale; SBP = systolic blood pressure.

Ethnic origin was self-reported by patients or families at the time of stroke unit admission and extracted from the patients' electronic health records as documented in the hospital database.

a Statistically significant results.

### Brain Imaging Characteristics

The brain imaging features of our study population are presented in Table 2. The ICH location was most commonly deep (162, 47.4%) and most frequently caused by arteriolosclerosis (131, 38.3%). No differences in ICH location or etiology were found between the DWI+ and DWI− groups.

**Table 2:** Brain Imaging Characteristics

Variables	All (N = 342)	DWI+ (N = 74)	DWI− (N = 268)	*p* Value
ICH volume (mL), median (IQR)	9 (3–23)	14 (5–36)	7 (2–20)	0.0006^a^
DWI-positive lesion distribution, n (%)				
DWI strictly lobar	—	33 (44.6)	—	—
DWI strictly deep	—	15 (20.3)	—	—
DWI strictly infratentorial	—	5 (6.7)	—	—
DWI mixed	—	21 (28.4)	—	—
DWI anatomical rating, n (%)				
Cortex	—	24 (32.4)	—	—
White matter	—	39 (52.7)	—	—
Basal ganglia	—	4 (5.4)	—	—
Thalami	—	0	—	—
Cerebellum	—	3 (4.0)	—	—
Brainstem	—	4 (5.4)	—	—
Mixed group^b^	—	10 (13.5)	—	—
ICH location, n (%)				
Lobar	138 (40.3)	33 (44.6)	106 (39.5)	0.378
Deep	162 (47.4)	32 (43.2)	129 (48.1)	—
Infratentorial	32 (9.4)	6 (8.1)	26 (9.7)	—
Mixed	10 (2.9)	3 (4.0)	7 (2.6)	—
ICH etiology, n (%)				
Arteriolosclerosis	131 (38.3)	33 (44.6)	98 (36.6)	0.209
Probable CAA	64 (18.7)	13 (17.6)	51 (19.0)	0.775
Mixed SVD	126 (36.8)	27 (36.5)	99 (36.9)	0.831
Cryptogenic (no SVD)	21 (6.1)	1 (1.3)	20 (7.5)	0.607
Individual SVD markers				
WMH, Fazekas ≥2, n (%)	232 (67.8)	60 (81.1)	172 (64.2)	<0.001^a^
WMH, Fazekas, median (IQR)	2 (1–3)	2 (2–3)	2 (1–3)	0.010^a^
CMB presence, n (%)	205 (59.9)	53 (71.6)	152 (56.7)	0.349
Total CMBs, median (IQR)	2 (0–7)	3 (0–9)	1 (0–5)	0.0342^a^
Lobar CMBs, median (IQR)	1 (0–5)	2 (1–6)	0 (0–3)	0.0451^a^
Deep CMBs, median (IQR)	0 (0–2)	0 (0–2)	0 (0–1)	0.0504
Infratentorial CMBs, median (IQR)	0 (0–1)	0 (0–2)	0 (0–1)	0.0528
cSS presence, n (%)	36 (10.5)	9 (12.2)	27 (10.1)	0.663
Disseminated cSS, n (%)	10 (2.9)	6 (8.1)	4 (1.5)	0.213
Lacune presence, n (%)	134 (39.2)	37 (50.0)	97 (36.2)	0.114
Total lacunes, median (IQR)	0 (0–1)	0 (0–2)	0 (0–1)	0.0499^a^
EPVS presence, n (%)	286 (83.62)	67 (90.54)	219 (81.72)	0.858
Total EPVS, median (IQR)	4 (2–5)	4 (3–5)	4 (2–5)	0.6574
Total SVD burden, median (IQR)	2 (1–3)	3 (2–4)	2 (1–3)	0.0423^a^

Abbreviations: CAA = cerebral amyloid angiopathy; CMB = cerebral microbleed; cSS = cortical superficial siderosis; DWI = diffusion-weighted imaging; EPVS = enlarged perivascular spaces; ICH = intracerebral hemorrhage; IQR = interquartile range; SVD = small vessel disease; WMH = white matter hyperintensities.

a Statistically significant results.

b Mixed group refers to patients with lesions affecting multiple anatomical regions, including overlap with other lesion categories.

**Figure 1:**
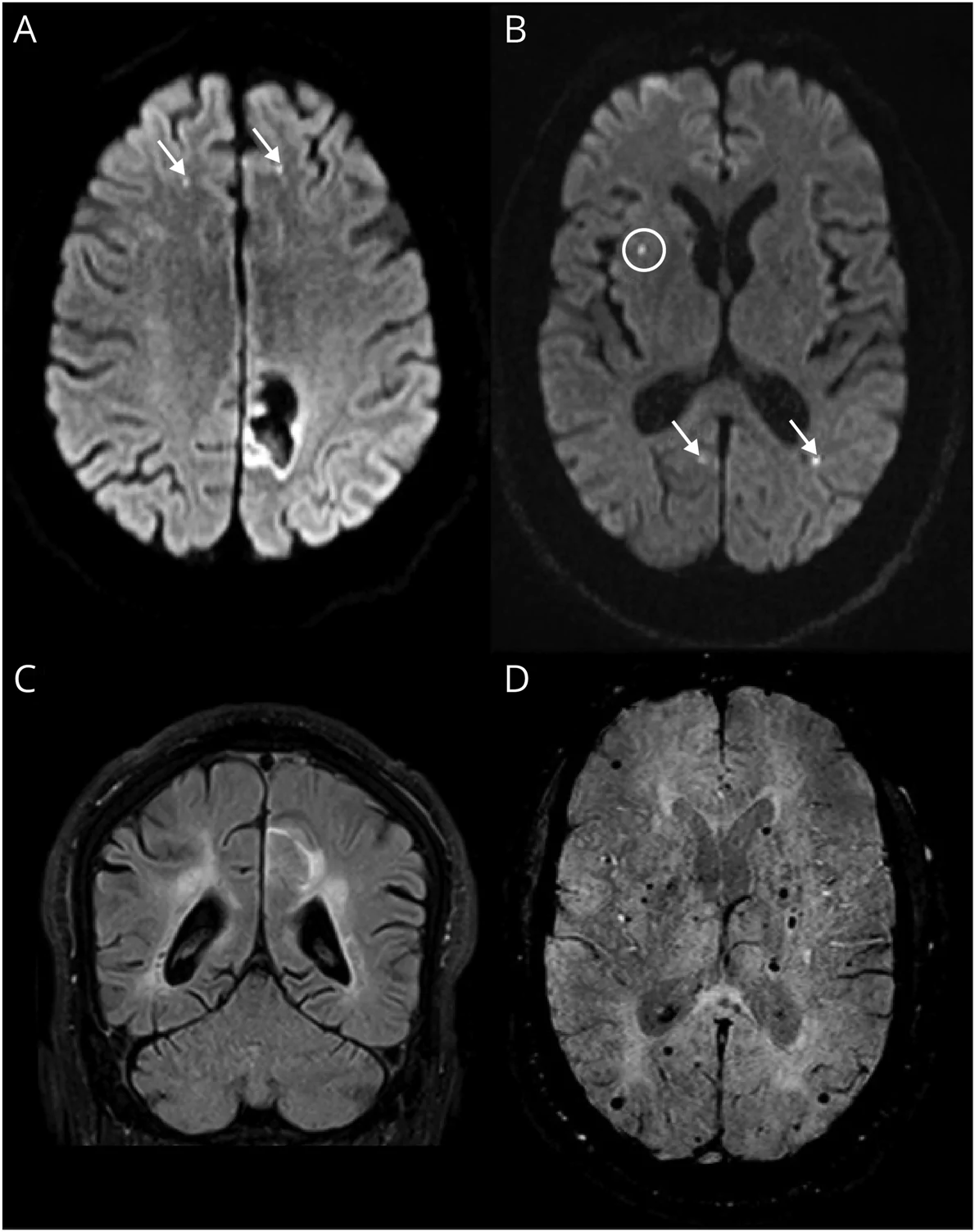
MRI Findings in a Patient Admitted Because of a Lobar Intracranial Hemorrhage *T2-weighted FLAIR MRI from a representative patient: (A) DWI sequences showing a left parietal hematoma centered on the precuneus and 2 foci of restricted diffusion in the left and right superior frontal gyri (arrows). (B) DWI sequences showing a deep right putaminal DWI-positive lesion (circle) and 2 additional lobar DWI-positive lesions in the right temporal lobe and left peritrigonal white matter (arrows). (C) FLAIR sequence showing early confluent periventricular white matter hyperintensities (Fazekas grade 2). (D) SWI sequences showing innumerable microbleeds, both lobar and deep. DWI = diffusion-weighted imaging; FLAIR = fluid-attenuated inversion recovery; SWI = susceptibility-weighted imaging.

Among patients with DWI-positive lesions (n = 74), 38/74 (51.4%) had a single lesion, 24/74 (32.4%) had 2–3 lesions, 7/74 (9.5%) had 4–5 lesions, and 5/74 (6.8%) had ≥6 lesions. DWI-positive lesions were most frequently located in the white matter (39, 52.7%) and were predominantly confined to lobar regions (33, 44.6%). Compared with the DWI− group, the DWI+ group had larger median ICH volume (14 mL [IQR 5–36] vs 7 mL [IQR 2–20]), a higher proportion of patients with severe WMH (81.1% vs 64.2%), slightly more lacunes (median [IQR] 0 [0–2] vs 0 [0–1]), and more CMBs (median [IQR] 3 [0–9] vs 1 [0–5]). No significant differences were observed regarding the prevalence of cSS or EPVSs between the 2 groups. Patients with DWI-positive lesions had a higher median combined total SVD burden score (3 [IQR 2–4] vs 2 [IQR 1–3]); an example is shown in Figure 1.

### Factors Associated With DWI-Positive Lesions

In the adjusted multivariable analysis, history of stroke/TIA (OR 1.84, 95% CI 1.47–3.37, *p* < 0.001), ICH volume >30 mL (OR 2.29, 95% CI 1.76–3.42, *p* < 0.001), Fazekas ≥2 WMH (OR 3.37, 95% CI 2.72–6.89, *p* < 0.001), and the presence of ≥5 CMBs (OR 1.61, 95% CI 1.21–2.93, *p* = 0.007) were independently associated with the presence of DWI-positive lesions (Table 3), and further adjusted analysis is presented in eTable 2.

**Table 3:** Multivariable Logistic Regression of Factors Associated With DWI-Positive Lesions

Variables	OR	95% Cl	*p* Value
Sociodemographic			
Age ≥60 y	0.70	0.37–1.32	0.231
Female sex	0.63	0.37–1.15	0.178
Previous stroke/TIA	1.84	1.47–3.37	<0.001^a^
Smoking	1.12	0.71–1.19	0.429
Alcohol	1.09	0.87–1.21	0.386
Brain imaging			
ICH volume ≥30 mL	2.29	1.76–3.42	<0.001^a^
WMHs, Fazekas ≥2	3.37	2.72–6.89	<0.001^a^
CMBs ≥5	1.61	1.21–2.93	0.007^a^
Lacunes	1.08	0.78–1.69	0.348
EPVS	1.06	0.82–1.15	0.709
CAA	0.97	0.63–1.49	0.763

Abbreviations: CAA = cerebral amyloid angiopathy; CMB = cerebral microbleed; DWI = diffusion-weighted imaging; EPVS = enlarged perivascular space; ICH = intracerebral hemorrhage; OR = odds ratio; SVD = cerebral small vessel disease; WMH = white matter hyperintensities.

a Statistically significant results.

### Distribution of DWI-Positive Lesions According to ICH Etiology

There was no difference in the overall prevalence of DWI+ lesions between patients with and without CAA, as presented in Table 4. However, patients with CAA more frequently had strictly lobar DWI-positive lesions compared with those without CAA (78.6% vs 36.7%), whereas strictly deep DWI-positive lesions were exclusively observed in the non-CAA group (0% vs 25.0%). The prevalence of strictly infratentorial DWI-positive lesions was similar between the 2 groups (7.1% vs 6.7%). These findings are summarized in Table 4.

**Table 4:** DWI-Positive Lesion Distribution According to Underlying Arteriopathy (CAA vs Non-CAA)

Variables	CAA (N = 64)	Non-CAA (N = 278)	*p* Value
DWI presence, n (%)	14 (23.3)	60 (21.6)	0.775
Distribution, n (%)			
DWI strictly lobar, n (%)	11 (78.6)	22 (36.7)	0.034^a^
DWI strictly deep, n (%)	0	15 (25.0)	<0.001^a^
DWI strictly infratentorial, n (%)	1 (7.1)	4 (6.7)	0.981
DWI mixed, n (%)	2 (14.3)	19 (31.7)	0.021^a^
DWI anatomical rating, n (%)			
Cortex	5 (35.7)	19 (31.7)	0.493
White matter	8 (57.1)	31 (51.7)	0.203
Basal ganglia	0	4 (6.7)	<0.001^a^
Thalami	—	—	—
Brainstem DWI	0	4 (6.7)	<0.001^a^
Cerebellum DWI	1 (7.1)	2 (3.3)	0.342

Abbreviations: CAA = cerebral amyloid angiopathy; DWI = diffusion-weighted imaging.

Mixed group: patients with lesions in multiple regions (overlapping with other categories).

a Statistically significant results.

### Associations Between DWI-Positive Lesions and Clinical Outcomes

Follow-up data were available for 332 patients. In univariable analysis, patients with DWI-positive lesions had worse functional outcomes compared with the DWI− group (Table 1) at discharge (median mRS 3 [IQR 1–4] vs 2 [IQR 1–4]) and after 6 months (median mRS 3 [IQR 2–5] vs 2 [IQR 1–3]). Moreover, the DWI group had a higher rate of stroke recurrence at 6 months (20.3% vs 6.3%). Both ischemic stroke (6.8% vs 3.4%) and recurrent ICH (13.5% vs 3.0%) were more frequent in patients with DWI-positive lesions. The mortality rate was nearly 3 times higher in the DWI+ group (13.5% vs 4.8%), although this was not statistically significant at the 5% level.

**Figure 2:**
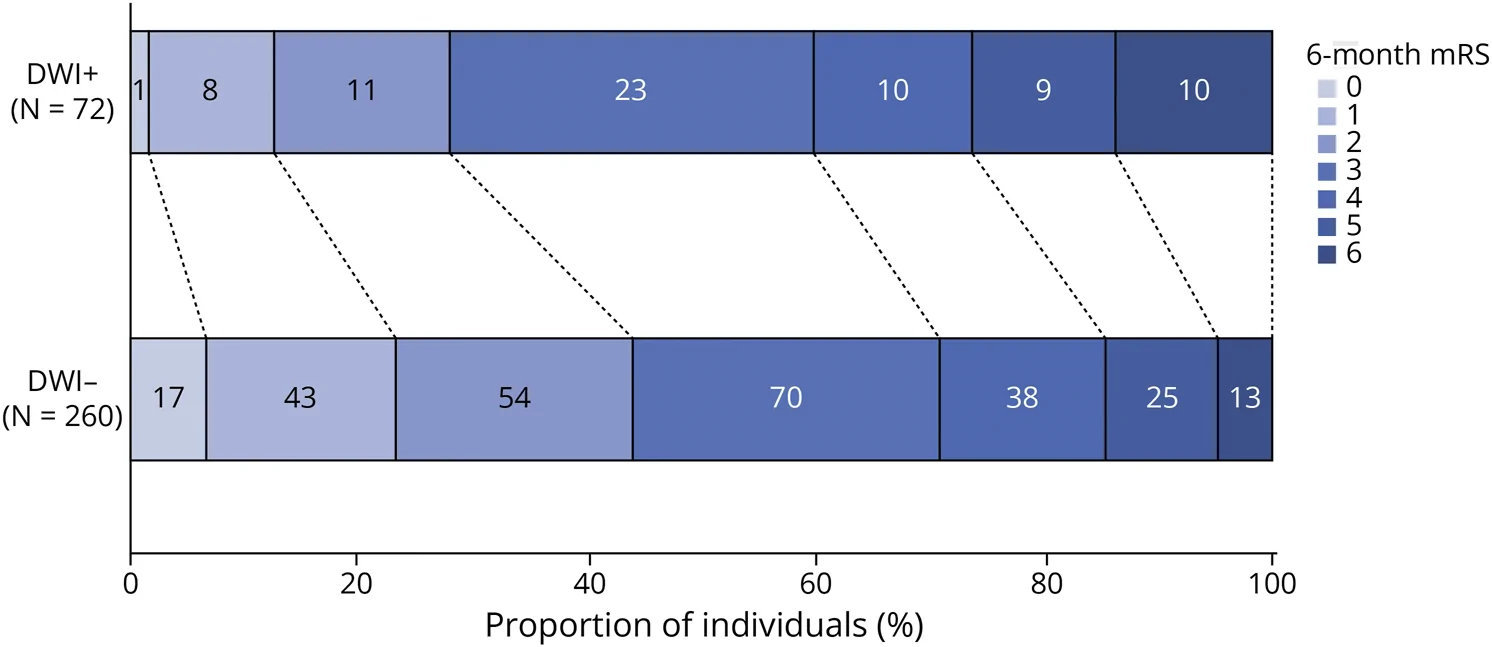
mRS at the 6-Month Follow-Up in DWI+ and DWI− Groups DWI = diffusion-weighted imaging; mRS = modified Rankin Scale. Six-month follow-up data were unavailable for 10 patients (2 DWI positive and 8 DWI negative).

Patients with poor 6-month outcomes (mRS 3–6, n = 198) were more likely to have DWI-positive lesions (26.26% vs 14.92%), as shown in Figure 2. The results of the unadjusted results are presented in eTables 3 and 4. In the adjusted analysis (Table 5, eTables 5 and 6), the presence of DWI-positive lesions was also associated with unfavorable outcome (OR 2.16, 95% 1.67–3.48, *p* < 0.001).

**Table 5:** Multivariable Logistic Regression of Factors Associated With Poor Clinical Outcome (mRS ≥3 at 6 Months)

Variables	OR	95% CI	*p* Value
DWI-positive lesions	2.16	1.67–3.48	<0.001^a^
Sociodemographic			
Age (ref <60 y)	1.36	1.10–1.74	0.005^a^
Female sex	1.06	0.60–1.59	0.749
Previous stroke/TIA	1.66	1.28–2.03	0.001^a^
Admission NIHSS ≥5	2.46	1.86–3.38	<0.001^a^
Hypertension	0.71	0.53–1.34	0.159
Diabetes	0.90	0.37–1.71	0.726
Premorbid mRS, median (IQR) ≥1	0.78	0.52–1.14	0.218
Brain imaging			
Lobar ICH location	1.08	0.51–1.93	0.821
CAA	1.41	0.68–2.94	0.449
WMH	2.46	1.71–4.06	<0.001^a^
CMBs ≥5	1.43	0.73–2.87	0.301
ICH Volume ≥30 mL	2.12	1.32–3.47	0.003^a^

Abbreviations: CAA = cerebral amyloid angiopathy; CMB = cerebral microbleed; DWI = diffusion-weighted imaging; ICH = intracerebral hemorrhage; IQR = interquartile range; mRS = modified Rankin Scale; NIHSS = NIH Stroke Scale; OR = odds ratio; WMH = white matter hyperintensities.

a Statistically significant results.

## Discussion

In our observational cohort of consecutive patients with ICH and available MRI, our major findings were that DWI-positive lesions (1) are common, being present in 21.6% of patients with ICH; (2) are associated with the presence and severity of neuroimaging markers of severe cSVD; (3) are not seen in deep locations (basal ganglia or brainstem) in patients with probable CAA; and (4) are independently associated with poor functional outcomes and recurrent stroke at the 6-month follow-up. These findings are consistent with the hypothesis that DWI-positive lesions reflect the severity of underlying SVD and have relevance for prognosis after ICH. Furthermore, the distribution of DWI-positive lesions may provide insights into the type of underlying small vessel arteriopathy.

The mechanisms underlying DWI-positive lesions in acute ICH remain uncertain. It has been suggested that they could be related to the acute ICH (e.g., neuroinflammation, disturbed autoregulation, and cortical spreading depression), subsequent therapy (e.g., early blood pressure lowering, surgery, or hemostatic therapy), the underlying small vessel arteriopathy that has caused the acute ICH, or a combination of some or all of these factors.^18,19,21^ Our findings of a high prevalence of DWI-positive lesions, which show strong associations with all MRI markers of cSVD and the total cSVD burden, suggest that incidental DWI-positive lesions are most likely due to the underlying vasculopathy. The associations of DWI-positive lesions with functional outcome and recurrent stroke that we have shown indicate that DWI-positive lesions could be a relevant neuroimaging biomarker for future clinical trials, including to select patients with active disease with a higher likelihood of clinical progression.

The hypothesis of DWI-positive lesions as a prognostic marker stems from their reflection of underlying SVD severity and tissue injury progression.^21^ A recent study observed that patients with DWI-positive lesions exhibit a greater increase in WMH volume over a 13-year follow-up, indicating faster SVD progression.^30^ The evolution of DWI-positive lesions into lacunes or WMHs has also been demonstrated, thereby contributing to SVD progression.^31,32^ In addition, pathologic studies suggest that even when a single DWI-positive lesion is visible on imaging, the corresponding tissue may contain thousands of subclinical microinfarcts, reflecting the accumulation of ischemic tissue damage over time.^33^

Our main finding was that functional outcome at 6 months was worse in patients with DWI-positive lesions, and the presence of DWI-positive lesion is independently associated with poor outcome, with a two-fold increased risk of mRS ≥3. Furthermore, patients with DWI-positive lesions more frequently experienced ischemic stroke or hemorrhagic recurrence within 6 months, compared with patients without DWI-positive lesions. No association was found between DWI-positive lesions and mortality. These findings are consistent with existing literature^34^ and expand current knowledge by providing follow-up beyond 3 months, unlike most previous studies.^16-19,35-37^

A history of stroke or TIA also independently predicted the presence of DWI-positive lesions, explained by patients with more advanced underlying SVD being more likely to have previously experienced symptomatic small vessel occlusion. No associations were observed with other vascular risk factors, including a history of hypertension or systolic blood pressure at admission, unlike previous studies.^15,19^ This discrepancy may be partially explained by the characteristics and methodological differences between our cohort and previous studies.

We also analyzed how the prevalence and distribution of DWI-positive lesions varied across etiological groups. Current evidence suggests that the prevalence of DWI-positive lesions does not significantly differ between arteriolosclerosis and CAA.^15^ This is not unexpected because both conditions involve pathologic changes in cerebral small vessels that can lead to hemorrhagic or ischemic events. Similarly, in our cohort, we did not observe any significant differences in the prevalence of DWI-positive lesions between the CAA and non-CAA groups. An important unresolved question is whether the distribution of DWI-positive lesions could help differentiate ICH etiological subtypes. Our findings suggest that the distribution of incidental DWI-positive lesions matches that of the underlying cSVD. Incidental DWI-positive lesions in CAA were confined to the lobar regions or cerebellum, in contrast to DWI-positive lesions in arteriosclerosis or mixed-pattern cSVD, which were also located in deep brain regions, including the basal ganglia and brainstem. We found that patients with CAA did not have DWI-positive lesions in the basal ganglia and brainstem. These findings could be explained by the predilection of CAA to affect predominantly superficial cortical perforating and leptomeningeal vessels. Our study suggests that the presence of deep DWI-positive lesions could have diagnostic relevance as an additional nonhemorrhagic diagnostic marker to exclude CAA. However, we acknowledge that the CAA/DWI-positive subgroup was very small (n = 14), so these findings should be interpreted cautiously and considered as hypothesis generating. Future studies in larger cohorts are needed to confirm this hypothesis.

Our study has several strengths. First, it includes a large consecutive sample, minimizing selection bias, and encompasses all ICH etiologies except secondary causes, as well as both supratentorial and infratentorial locations, which were often only partially represented in earlier research.^16,36,38^ In addition, the largest available studies evaluating a longer follow-up may have been affected by selection bias because of their randomized nature^20,39^ or retrospective design^40^ and by heterogeneity in imaging protocols and patient management stemming from their multicenter settings.^20,39,41^ Second, unlike many previous studies that focused primarily on Asian populations,^16-18^ our cohort includes patients from multiple ethnic backgrounds, further enhancing the external validity of our findings. Third, DWI-positive lesions in close vicinity to the hemorrhage were excluded, thereby minimizing confounding effects related to local compression from the hematoma. In addition, the time from admission to MRI acquisition was comparable between DWI+ and DWI− groups, reducing potential bias in lesion detection related to timing. Furthermore, we are not aware of other studies that analyzed the distribution of DWI-positive lesions in detail as a potential marker of the underlying cSVD subtype. All raters were trained by the same expert neuroradiologist, and intrarater and inter-rater reliability coefficients were acceptable (κ > 0.75).

We also acknowledge some limitations. We excluded a proportion of patients because brain imaging on admission was unavailable, primarily because diagnostic MRI was unavailable or contraindicated. Another limitation is the lack of detailed data on hyperacute blood pressure–lowering therapy, which limits our ability to assess its potential impact on outcomes. The excluded individuals appeared to be more fragile and to have a worse prognosis, which may have introduced a selection bias and limited the generalizability of our findings to less clinically severe patients with ICH. Moreover, the observed absence of basal ganglia or brainstem DWI lesions among patients with probable CAA should be interpreted with caution, given the small sample size of the CAA subgroup. Therefore, larger studies are required to validate these findings.

In conclusion, our findings suggest that DWI-positive lesions have mechanistic, prognostic, and diagnostic clinical relevance in patients with acute ICH. Detecting these lesions may improve risk stratification by reflecting cSVD severity and identifying patients at higher risk of long-term disability and stroke recurrence, who may benefit from more aggressive and personalized management strategies aimed at optimizing functional outcomes or who could be selected for disease-modifying clinical trials.

## Glossary

ADC: apparent diffusion coefficient
BGPVS: basal ganglia perivascular spaces
CAA: cerebral amyloid angiopathy
CMB: cerebral microbleed
cSS: cortical superficial siderosis
cSVD: cerebral small vessel disease
DWI: diffusion-weighted imaging
EPVS: enlarged perivascular spaces
FLAIR: fluid-attenuated inversion recovery
ICH: intracerebral hemorrhage
MARS: Microbleed Anatomical Rating Scale
mRS: modified Rankin Scale
OR: odds ratio
SIGNAL: Stroke Investigation Group in North and Central London
STRIVE: STandards for ReportIng Vascular changes on nEuroimaging
SVD: small vessel disease
UCL: University College London
WMH: white matter hyperintensities
